# A Four‐Item Risk Score to Target Acute HIV Infection Testing Among Men Who Have Sex With Men in Indonesia: Development and Validation in the INTERACT Prospective Cohort

**DOI:** 10.1002/jia2.70111

**Published:** 2026-04-15

**Authors:** Gilbert Lazarus, Nurhayati H. Kawi, Hendry Luis, Dwi P. Rahmawati, Erik P. Sihotang, Margareta Oktaviani, Pande Putu Januraga, Suwarti, Evi Sukmaningrum, Evy Yunihastuti, Maartje Dijkstra, Eduard J. Sanders, Frank Stephen Wignall, Keerti Gedela, Irwanto, Raph L. Hamers, the INTERACT Study Group

**Affiliations:** ^1^ Oxford University Clinical Research Unit Indonesia Faculty of Medicine Universitas Indonesia Jakarta Indonesia; ^2^ Klinik Utama Globalindo Jakarta Indonesia; ^3^ Yayasan Bali Peduli Denpasar Indonesia; ^4^ Center for Public Health Innovation Udayana University Denpasar Indonesia; ^5^ Faculty of Psychology Atma Jaya Catholic University of Indonesia Jakarta Indonesia; ^6^ Dr Cipto Mangunkusumo Hospital Faculty of Medicine Universitas Indonesia Jakarta Indonesia; ^7^ Department of Internal Medicine Amsterdam University Medical Centers University of Amsterdam Amsterdam The Netherlands; ^8^ The Aurum Institute Johannesburg South Africa; ^9^ Sir William Dunn School of Pathology University of Oxford Oxford UK; ^10^ 56 Dean Street Clinic Chelsea & Westminster Hospital NHS Trust London UK; ^11^ Centre for Tropical Medicine and Global Health Nuffield Department of Medicine University of Oxford Oxford UK

**Keywords:** acute HIV infection, HIV, Indonesia, men who have sex with men, prediction model, risk score

## Abstract

**Introduction:**

Indonesia has an escalated HIV epidemic among key populations, especially men who have sex with men (MSM). Diagnosis and immediate treatment of acute HIV infection (AHI), the earliest phase with the highest transmission risk, is beneficial for individual health and can reduce onward transmission. To inform whom to test for possible AHI using targeted, risk‐stratified HIV‐PCR testing, this study evaluated the performance of the validated, seven‐item Amsterdam AHI risk score among Indonesian MSM, and developed a locally optimized score.

**Methods:**

We used the INTERACT prospective cohort of MSM (≥16 years) attending sexual health clinics in Jakarta and Bali (May 2023−February 2025) who were tested with add‐on Xpert HIV‐PCR (Cepheid) if their HIV antibody rapid testing was negative or inconclusive. We used generalized estimating equation models to generate risk scores, combining symptoms, risk factors and socio‐demographics. The optimized risk score was internally validated using bootstrap resampling. We calculated area under the curve (AUC), sensitivity and specificity (ISRCTN41396071).

**Results:**

Among 1887 individuals, 20 were diagnosed with AHI, and 1867 tested AHI negative across 3446 test visits. The Amsterdam score yielded an AUC of 0.82 (95% CI 0.75−0.90) with a sensitivity of 85.0% (64.0%−94.8%) and a specificity of 59.2% (57.5−60.8). The optimized risk score included one symptom (fever <2 weeks), one risk factor (condomless receptive anal intercourse <6 months) and two socio‐demographic characteristics (age 16–30 years, not having received higher education), and achieved an AUC of 0.91 (0.87−0.96) with a sensitivity of 100% (83.9−100) and a specificity of 65.3% (63.6%−66.8%). Internal validation yielded an AUC of 0.86 (0.67−0.97). Applying this risk score would classify 35.1% of MSM as eligible for add‐on HIV‐PCR testing, identifying 83.9%–100% of individuals who have AHI.

**Conclusions:**

This four‐item risk score of easily collected variables can facilitate efficient AHI detection in high‐yield clinic settings, enhancing opportunities for HIV prevention. In the Indonesian context, younger MSM with lower educational attainment were particularly vulnerable to AHI.

**Abbreviations**
AHI: acute HIV infection
AUC: area under the curve
CI: confidence interval
CRAI: condomless receptive anal intercourse
HIV: human immunodeficiency virus
HIV‐PCR: HIV polymerase chain reaction
INTERACT: Indonesia Intervention Study to Test & Treat People with Acute HIV Infection
MSM: men who have sex with men
PLWH: person(s) living with HIV
PTPD: post‐test probability of disease
PrEP: pre‐exposure prophylaxis for HIV
RDT: rapid diagnostic test
STI: sexually transmitted infection

## 1 Introduction

The Joint United Nations Programme on HIV/AIDS aims to end AIDS as a public health threat by 2030. The first 2–3 weeks after HIV acquisition—known as acute HIV infection (AHI)—are characterized by peak viral load and infectiousness, and absence of antibodies [1, 2]. Mathematical models suggest that 10%–50% of new HIV acquisitions arise from individuals with AHI [1, 3, 4], indicating that targeting this phase could support epidemic control. However, AHI is frequently missed because of its brief duration, non‐specific symptoms, limited awareness among key populations and healthcare providers [5], and poor sensitivity of standard antibody‐based rapid diagnostic tests (RDTs) [6, 7, 8]. Although point‐of‐care HIV‐PCR tests can sensitively detect AHI, their use is constrained by cost, complexity and uncertainty about whom to test. Incorporating same‐day AHI “test‐and‐treat” pathways in sexual health clinics could strengthen treatment‐as‐prevention efforts [1, 9, 10].

Indonesia, a diverse middle‐income country of 280 million people, had an estimated 570,000 people living with HIV (PLWH) and 28,000 individuals newly acquired HIV in 2023 [11, 12]. Men who have sex with men (MSM) accounted for nearly one‐third of new HIV acquisitions [13, 14]. Stigma, economic constraints and limited access to pre‐exposure prophylaxis (PrEP) contribute to persistent gaps across the care cascade [15, 16]. HIV‐PCR testing for AHI detection is not included in the national HIV programme.

AHI risk score algorithms based on clinical, behavioural and socio‐demographic factors can improve efficiency by targeting scarce HIV‐PCR resources, while identifying a substantial proportion of individuals with AHI [17]. Such tools have been developed for MSM attending sexual health services in the United States, The Netherlands, Malawi and Kenya [17, 18, 19, 20]. However, implementation beyond study settings remains limited, and transferability may be constrained by differences in clinical presentation, risk behaviours and healthcare‐seeking.

To address this gap, the Indonesia Intervention Study to Test and Treat persons with AHI (INTERACT) implemented an AHI‐focused same‐day “test‐and‐treat” care pathway at sexual health clinics in Jakarta and Bali [8]. The intervention combined a digital AHI risk self‐assessment tool, add‐on point‐of‐care HIV‐PCR testing and same‐day antiretroviral therapy (ART) initiation. This analysis evaluated the performance of the Amsterdam AHI risk score [18, 21, 22], and developed a locally optimized risk score to identify MSM with possible AHI in Indonesia.

## 2 Methods

### 2.1 Study Population

INTERACT was a prospective cohort at three high‐volume, non‐governmental sexual health clinics in Jakarta and Bali, provinces with the highest HIV prevalence (behind Papua) [8]. Briefly, we consecutively recruited consenting adults (≥16 years) presenting for voluntary HIV testing, not known to be living with HIV, between May 2023 and February 2025. Participants were encouraged to return for quarterly AHI testing, or earlier in case of perceived AHI risk or symptoms. Participant blood specimens that were either negative on fourth‐generation antibody/p24 antigen (4gRDT; Abbott Determine HIV Early Detect) screening, or discordant with third‐generation RDT (Bioline HIV1/2, or equivalent) confirmatory testing, were additionally tested with Xpert HIV‐1 Qual assay (Cepheid) [8]. AHI was pragmatically defined as antibody‐negative or ‐discordant RDTs with a positive Xpert HIV‐PCR. Participants newly diagnosed with HIV (including AHI) were offered same‐day antiretroviral treatment initiation.

The Atma Jaya Catholic University of Indonesia research ethics committee (0009R/III/PPPE.PM.10.05/10/2022) and the Oxford Tropical Research Ethics Committee (565‐22) approved the study. Each participant provided written consent.

### 2.2 Participant Questionnaire

Participants completed a questionnaire in REDCap at each visit, including the Amsterdam Score [18, 21, 22]. Guided by the literature [17, 22, 23] and expert consultation, we captured data on socio‐demographics, risk behaviour, sexually transmitted infections (STIs), other symptoms and PrEP use. The questionnaire was translated from English to Indonesian and back‐translated.

### 2.3 Performance of the Validated Amsterdam AHI Score

We evaluated the performance of the seven‐item Amsterdam Score which has demonstrated good performance across several MSM populations [18, 21, 22], using published point values (weights); comprising four symptoms (fever [1.6], lymphadenopathy [1.5], weight loss [0.9], oral thrush [1.7], each <2 weeks) and three risks (self‐reported gonorrhoea [1.6], >5 sexual partners [0.9], condomless receptive anali intercourse (CRAI) [1.1], each <6 months) [18, 21, 22].

### 2.4 Development and Validation of the Optimized AHI Risk Score

We assessed 60 independent variables for their association with AHI, using generalized estimating equations with an exchangeable covariance matrix and robust standard errors to account for repeated measures (Table 1). Missing data for education level (*n* = 12) were handled via complete‐case analysis or by coding missing as “no higher education” (conservative analysis). Independent variables with *p*≤0.10 in univariable analysis were included in multivariable models, with selection guided by quasi‐information criteria and highest AUC. Results were expressed using odds ratios (ORs) with 95% confidence intervals (CIs) and two‐sided *p*‐values (*p*<0.05 statistically significant). Subsequently, we created a score with each variable weighted as the beta‐coefficient (β) in the multivariable model [18, 21, 22]. Model performance was assessed using AUC, sensitivity and specificity. We generated four receiver operating characteristic curves for: (i) Amsterdam Score [18, 21, 22]; local risk score with (ii) complete‐case analysis (main model); (iii) missing values retained; and (iv) complete‐case analysis using a broader definition of AHI (also including seroconversion, i.e. antibody‐positive RDT following a negative test <6 months prior [8]). Optimal cut‐off scores were defined by the Youden index, which provides an objective measure of overall accuracy by identifying a threshold that best balances sensitivity and specificity [24]. Efficiency was estimated as the proportion of visits to be tested (number of visits scoring above the cut‐off divided by the total number of visits). For internal validation, we performed bootstrap resampling with 5000 replicates. In each sample, the entire predictor selection and model fitting process was repeated, and optimism‐corrected discrimination and calibration were obtained by comparing performance in the bootstrap and original datasets. Optimism was calculated as the average difference in discrimination (AUC) and calibration slope, then subtracted to yield optimism‐corrected estimates. Regression coefficients were adjusted with a uniform shrinkage factor to reduce overfitting [25].

**TABLE 1:** Variables included in the final multivariable AHI prediction model.

				Multivariable analysis		Data completeness	
Variable	Total (*n* = 3682)	Persons with AHI (*n* = 21)	Persons who tested AHI negative (*n* = 3661)	Odds ratio (95% CI)	*p*‐value	*N*	Missing (%)
Fever in the last 2 weeks	647 (18.8%)	14 (70.0%)	633 (18.5%)	8.6 (3.2−22.5)	<0.001	3446	0 (0.0%)
Condomless receptive anal intercourse in the last 6 months	821 (22.4%)	12 (57.1%)	809 (22.2%)	15.8 (2.1−119.8)	0.008	3446	0 (0.0%)
Not received higher education	766 (22.3%)	12 (60.0%)	754 (22.1%)	3.5 (1.4−8.7)	0.007	3434	12 (0.35%)
Age <30 years	2018 (58.6%)	19 (95.0%)	1999 (58.3%)	7.8 (1.0−58.6)	0.047	3446	0 (0.0%)

*Notes*: The table presents the results of the multivariable prediction model of symptoms, risk factors and socio‐demographics predictive of AHI. Odds ratios are derived from generalized estimating equations, with an exchangeable covariance matrix and robust standard errors to adjust for multiple observations per participant.

Variables considered in the univariable analysis and found not to be associated (i.e. *p*>0.10 or not estimable) were: (i) socio‐demographics: marital status; (ii) risk: receptive anal intercourse, condomless receptive anal intercourse with a PLWH, genital discharge or ulcer (all in the last 6 months); group sex, sex parties, intravenous drug use, chemsex (all in the last 3 months); multiple sex partners; being a sex worker client, sex worker or transgender person; sex with a PLWH; (iii) symptoms/clinical: current or previous (<12 months) STI; current positive syphilis serology (defined as a reactive RPR and treponemal RDT); current's clinician diagnosis of syphilis; current gonorrhoea, chlamydia, hepatitis B, hepatitis C or genital herpes; oral thrush; sore throat; skin rash; lymphadenopathy; vomiting; mouth blisters; nausea; flu‐like symptoms; joint pain; diarrhoea; genital warts; genital blisters (all in the last 2 weeks); and (iv) prior or recent PrEP use.

Variables considered in the multivariable model (*p*≤0.10 in univariable analysis) and found not to be associated (i.e. *p*≥0.05 in the multivariable model) were night sweats, weight loss, fatigue, sore throat, headache, muscle pain (all in the last 2 weeks); condom use; incident syphilis infection (defined as seroconversion from non‐reactive RPR to reactive RPR+treponemal RDT); having a fixed partner; and being a student.

Abbreviations: AHI, acute HIV infection; PLWH, person living with HIV; PrEP, pre‐exposure prophylaxis of HIV; RDT, rapid diagnostic test; RPR, rapid plasma regain; STI, sexually transmitted infection.

### 2.5 Post‐Test Probability of Disease

To evaluate the clinical relevance of the optimized risk score, we estimated the post‐test probability of disease (PTPD; i.e. the likelihood of having AHI when having a score above the risk score cut‐off), using a pre‐test probability of 0.72% [8].

All analyses were performed using Stata 17.0 (StataCorp, Texas, US).

## 3 Results

### 3.1 Participant Characteristics

The analysis included a total of 3738 visits from 2179 individuals (1697 from Jakarta and 482 from Bali). Median age at enrolment was 28 years (IQR 25−32). One thousand four hundred and three (64.4%) individuals had one visit only (range 1–10). Returning individuals (828, 35.5%) had a median follow‐up of 8 months (IQR 4−14). Individuals reported recent (<1 month prior) PrEP use at 10.0% (372/3717) of visits, with no AHI detected among PrEP users. Twenty individuals were diagnosed with AHI, and 1867 individuals tested AHI negative across 3446 visits. The AHI incidence rate was 2.7 (95% CI 1.7−4.2) per 100 person‐years (total time‐at‐risk 8891 months), and higher in Bali (4.5 [2.2−8.9]) compared to Jakarta (2.1 [1.2−3.8] (*p* = 0.075), although this was not statistically significantly different.

### 3.2 Performance of the Validated Amsterdam AHI Score

**FIGURE 1:**
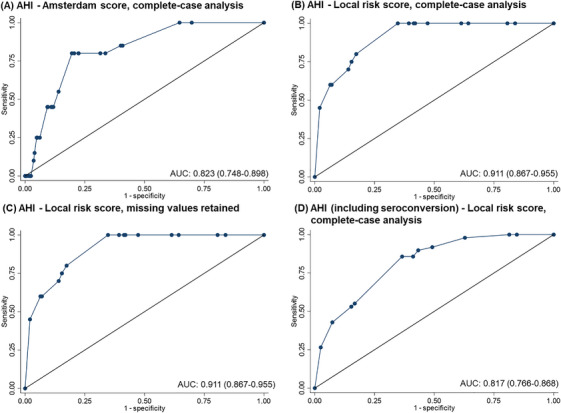
Receiver operating characteristic curves of risk scores for predicting AHI in men who have sex with men in Indonesia: (A) Amsterdam score, (B) local risk score, complete‐case analysis, (C) local risk score, missing values retained, (D) local risk score, complete‐case analysis predicting AHI (including HIV seroconversion). The Amsterdam AHI Score (A) included the following seven variables: fever, swollen lymph nodes, weight loss, oral thrush, each within the past 2 weeks; gonorrhoea, >5 sexual partners, CRAI, each within the past 6 months. Each local AHI risk score (B−D) included the following four variables: fever in the last 2 weeks, CRAI in the last 6 months, age 16–30 years old and no higher education received. In (C), values for education level that were “missing” were retained with their values coded as “1” (meaning “did not receive higher education”) to produce a more conservative analysis. Little's MCAR (missing completely at random) tests and missing‐indicator analysis by regression analysis did not identify bias of missing education level data with respect to AHI. In (D), the local risk score was applied to AHI, including participants with HIV seroconversion, as the outcome variable, using complete‐case analysis. Abbreviations: AHI, acute HIV infection; AUC, area under the curve; CRAI, condomless receptive anal intercourse.

The Amsterdam Score (cut‐off 1.5) yielded an AUC of 0.82 (95% CI 0.75−0.90) with a sensitivity of 85.0% (64.0%−94.8%) and a specificity 59.2% (57.5−60.8), rendering 41.1% (1415/3446) of the visits indicated for HIV‐PCR testing (Figure 1 and Table 2).

**TABLE 2:** Performance of AHI risk scores in predicting acute HIV infection (AHI) in men who have sex with men in Indonesia.

		Visits with persons testing positive for AHI					
Risk score (point values)	Cut‐off^a^	Risk score ≥ cutoff	Risk score < cutoff	Sensitivity (95% CI)	Specificity (95% CI)	AUC (95% CI)	Percentage visits to be HIV‐PCR tested
**(A)** Amsterdam score, complete‐case analysis, predicting AHI: Fever (β 1.6) + Swollen lymph nodes (β 1.5) + Weight loss (β 0.9) + Gonorrhoea in the last 6 months (β 1.6) + CRAI in the last 6 months (β 1.1) + Number of partners ≥5 (β 0.9)^b^, ^c^	1.5	17/1415	3/2031	85.0% (64.0−94.8)	59.2% (57.5−60.8)	0.823 (0.748−0.898)	41.1%
**(B)** Local risk score, complete‐case analysis, predicting AHI: Fever in the last 2 weeks (β 2.1) + CRAI in the last 6 months (β 2.8) + Not received higher education (β 1.3) + Age 16–30 years (β 2.1)^d^	4.5	20/1206	0/2228	100% (83.9−100)	65.3% (63.6−66.8)	0.911 (0.867−0.955)	35.1%
**(C)** Local risk score, missing values retained, predicting AHI: Fever in the last 2 weeks (β 2.1) + CRAI in the last 6 months (β 2.8) + Not received higher education (β 1.3) + Age 16–30 years (β 2.1)^e^	4.5	20/1209	0/2237	100% (83.9−100)	65.3% (63.6−66.8)	0.911 (0.867−0.955)	35.1%
**(D)** Local risk score, complete‐case analysis, predicting AHI (including HIV seroconversion): Fever in the last 2 weeks (β 2.1) + CRAI in the last 6 months (β 2.8) + Not received higher education (β 1.3) + Age 16–30 years (β 2.1)^f^	4.5	42/1371	7/2314	85.7% (73.3−92.9)	63.4% (61.9−65.0)	0.817 (0.766−0.868)	37.2%

*Note*: Risk scores were modelled using generalized estimating equations, with an exchangeable covariance matrix and robust standard errors to adjust for multiple observations per participant. Each item in the risk score was scored as a beta‐coefficient in a multivariable model.

Abbreviations: AHI, acute HIV infection; AUC, area under the receiver operating characteristic curve; CRAI, condomless receptive anal intercourse.

^a^ Cut‐off levels were defined by each possible sum of the score of symptoms and risk factors, based on the beta coefficient. The local risk score used a cut‐off of 4.5 points, which was selected based on the Youden index; the Amsterdam AHI risk score used their original cut‐off of 1.5 points.

^b^ Adopted from the Amsterdam AHI Score [18].

^c^ Risk score was tested on 3446 visits.

^d^ Risk score was developed based on 3434 visits (12 missing values for education level).

^e^ Risk score was developed based on 3446 visits (no missing values). Twelve missing values for education level was retained by imputing them to “1” (no higher education) to produce a more conservative analysis, considering that all participants with missing education level data were AHI negative and that the data were deemed to be missing at random as Little's MCAR (missing completely at random) test and missing‐indicator analysis by regression analysis did not identify bias of missing education level data with respect to AHI.

^f^ This analysis used a broader definition of AHI (including HIV seroconversion). Risk score was developed based on 3685 visits (12 missing values for education level).

### 3.3 Development and Validation of the Locally Optimized Risk Score

In the multivariable model, four variables were statistically significantly associated with AHI diagnosis: fever <2 weeks (odds ratio 8.5 [95% CI 3.2−22.5], *p*<0.001), CRAI <6 months (15.8 [2.1−119.8], *p* = 0.008), age 16–30 years old (7.8 [1.0−58.6], *p* = 0.047) and not having received higher education (3.5 [1.4−8.7], *p* = 0.007) (Table 1). At a Youden‐defined cut‐off of 4.5, both the complete‐case analysis and the analysis with missing values retained yielded an AUC of 0.91 (0.87−0.96), a sensitivity of 100% (83.9%–100%) and a specificity of 65.3% (63.6%−66.8%), rendering 35.1% of the visits indicated for testing (Figure 1 and Table 2). Internal validation yielded good discrimination and calibration with a shrinkage factor of 0.974 and an optimism‐corrected AUC of 0.86 (0.67−0.97). Across the 5000 bootstrap samples, the inclusion frequency was highest for fever (95.6%), followed by education (65.3%), CRAI (56.0%) and age (17.3%). The PTPD was 2.0% (1.8%−2.1%), and the positive likelihood ratio was 2.9 (2.7−3.0).

## 4 Discussion

This analysis is among the first in Asia to develop an AHI risk score for MSM attending sexual health services [17]. The good performance of a simple four‐item score—reported fever, CRAI, young age and low educational attainment—suggests that easily collected variables can effectively target AHI testing in clinic settings. In our cohort, the seven‐item Amsterdam Score performed less well than the locally optimized score. Our findings are consistent with previously identified AHI predictors among non‐Asian MSM populations, including young age, multiple partners, CRAI, sex with a PLWH, self‐reported STI and illicit drug use, as well as symptoms such as diarrhoea and fever [17, 26, 27, 28]. Differences in variable selection and performance likely reflect variation in clinical presentation, risk behaviour, socio‐demographics and healthcare‐seeking. This analysis confirmed the high HIV incidence (2.7 per 100 person‐years) among MSM in Jakarta and Bali [12, 13].

Selection of risk score cut‐offs depends on balancing sensitivity (minimizing false‐negatives), specificity (minimizing false‐positives) and HIV‐PCR testing costs [1, 25]. Risk‐stratified targeted AHI testing can reduce test costs while maintaining high case detection [17, 29]. In our setting, the optimized risk score would classify 35.1% of MSM seeking standard testing as eligible for add‐on HIV‐PCR and identify 83.9%–100% of individuals with AHI. Several published AHI risk scores lack validation [26, 27, 28] or showed reduced performance in validation cohorts [22, 30, 31]. Because of the limited number of persons with AHI in our cohort, we used the full dataset for model development; internal validation showed only a modest decrease in AUC (from 0.91 to 0.86). External validation in comparable, clinically relevant populations that reflects the risk score's intended use remains necessary.

Fever is a common feature of the acute retroviral syndrome [2]. Receptive anal intercourse substantially increases HIV acquisition risk [32], due to the susceptibility of the rectal mucosa [33]. Younger MSM bear a disproportionate burden of new HIV acquisitions in Asia and globally [34, 35, 36], driven by behavioural, socio‐economic and psychosocial factors including stigma, limited education and substance use [35]. Lower educational attainment has been associated with HIV acquisition among MSM, consistent with limited knowledge about HIV prevention, poor healthcare access and socio‐economic disadvantage [13, 37]. A previous study found higher HIV incidence among MSM who had not received higher education in Jakarta, but this was not the case in Bali [13]. In contrast with meta‐analyses demonstrating a two‐ to three‐fold increase in HIV acquisition following syphilis exposure [38], in our study, syphilis was not independently associated with AHI. Since treponemal and non‐treponemal antibodies develop 2–4 weeks after infection or later, the absence of syphilis seroreactivity at the time of AHI does not exclude concurrent syphilis infection, nor preclude a contributory role in facilitating HIV acquisition [39, 40]. Chlamydia and gonorrhoea have been variably associated with HIV acquisition, reflecting differences in local co‐epidemics and under‐detection due to low rates of STI testing [22, 28, 41, 42], as is the case in Indonesia [12].

We used HIV‐PCR rather than 4gRDT, because of the latter's low sensitivity for AHI detection in our setting [8], consistent with previous reports [1, 43]. Integrating add‐on HIV‐PCR screening into routine care for MSM testing seronegative could improve HIV‐PCR test acceptance and retention [8]. Further operational research and economic evaluations are needed to assess whether increased costs and complexity of HIV‐PCR testing are offset by HIV prevention benefits [29, 44, 45]. While implementing risk scores in clinical practice can be challenging [25], digital platforms offer scalable solutions. As part of INTERACT, we developed CekUpYuk.id, a community‐driven digital tool that includes an AHI self‐assessment tool [46]; this will be updated with the optimized risk score.

There are some limitations. Participants were MSM attending sexual health clinics in Jakarta and Bali, who may not represent other key populations, government community clinics or geographic settings. National survey data from 2023 estimated high HIV prevalence among MSM (24.4%), with one‐third reporting condomless anal sex and an average of 4–5 sex partners in the past 12 months, alongside persistent gaps in HIV testing, prevention and care, linked to stigma and avoidance of health services [12]. As PrEP rollout expands, HIV acquisition and testing patterns will shift; this was illustrated by the fact that among those using PrEP, no person with AHI was detected [47, 48]. Nevertheless, updated AHI risk scores will remain useful for targeting HIV‐PCR testing and preventing inadvertent PrEP initiation during undiagnosed AHI.

## 5 Conclusions

Prioritizing HIV‐PCR testing for MSM at the highest AHI risk could improve efficiency while enabling rapid interventions, including same‐day treatment and partner notification. In Indonesia, younger MSM and those with lower educational attainment were particularly vulnerable to AHI. Integrating AHI risk screening into HIV programmes could strengthen efforts to curb HIV transmission.

## Author Contributions

I, KG and RLH are the principal investigators. I, MD, FSW, KG and RLH conceptualized the study. I, NHK, HL, PPJ, FSW, MD, EJS, KG and RLH designed the study protocol. NHK, HL, S, DPR and MO established the cohort and collected the study data and samples. NHK, HL and S supervised the laboratory assays. NHK, HL, MO and DPR managed the clinical database and contributed to data verification. GL performed the statistical analyses and data visualizations, under the supervision of RLH. GL and RLH drafted the manuscript with critical contributions from PPJ, MD and EJS. GL and RLH had full access to all of the study data and took responsibility for the integrity of the data and the accuracy of the data analysis. All authors provided valuable input to the interpretation of the data and critically reviewed the paper and figures for important intellectual content. All authors reviewed and approved the final version of the manuscript.

## Author Information

**Indonesia Intervention Study to Test & Treat People with Acute HIV Infection (INTERACT) Study Group**:

Prof Irwanto (deceased; died on 27 May 2025), Ignatius Praptoraharjo, Arie Rahadi, **Evi Sukmaningrum** (Faculty of Psychology, Atma Jaya Catholic University of Indonesia, Jakarta)

Suwarti, Decy Subekti, Bachtiar Andy Mussafa, Nicolas Tarino, Mutia Rahardjani, Fitri Dewi, Soraya Weldina Ragil Dien, Margaret Oktavia, Dwi Rahmawati, Raph Hamers (Oxford University Clinical Research Unit Indonesia, Faculty of Medicine Universitas Indonesia, Jakarta)

**Keerti Gedela** (56 Dean Street, Chelsea & Westminster Hospital, Imperial College London, UK)

Prof **Pande Putu Januraga** (Center for Public Health Innovation, Udayana University, Bali)

Prof **Evy Yunihastuti** (Dept of Internal Medicine, Dr Cipto Mangunkusumo Hospital, Faculty of Medicine, Universitas Indonesia, Jakarta)

**Nurhayati H. Kawi**, **Erik P. Sihotang**, F. Stephen Wignall (Yayasan Globalindo, Jakarta)

**Hendry Luis**, F. Stephen Wignall (Yayasan Bali Peduli, Bali)

Godelieve de Bree, **Maartje Dijkstra** (Amsterdam UMC, location AMC, University of Amsterdam, Amsterdam, The Netherlands)

Prof Eduard Sanders (The Aurum Institute, Johannesburg, South Africa; and University of Oxford, Sir William Dunn School of Pathology, Oxford, UK)

Sayem Ahmed (University of Glasgow, Glasgow, UK)

Prof Christophe Fraser, Katrina Lythgoe (Big Data Institute, University of Oxford, Oxford, UK)

## Funding

This research is jointly funded by the UK Medical Research Council (MRC) and the Foreign Commonwealth and Development Office (FCDO) under the MRC/FCDO Concordat agreement (grant no. MR/V035304/1). RLH is supported by the Wellcome Africa Asia Programme Vietnam (106680/Z/14/Z).

## Conflicts of Interest

The authors declare no conflicts of interest.

## Clinical Study Registry

ISRCTN41396071

## Data Availability

Data are available upon reasonable request. Requests for data sharing can be made by submission of a study concept to the INTERACT Study Group for evaluation of the scientific value, relevance, design, feasibility and overlap with existing projects.
